# A Critical Evaluation of Interlaboratory Data on Total, Elemental, and Isotopic Carbon in the Carbonaceous Particle Reference Material, NIST SRM 1649a

**DOI:** 10.6028/jres.107.022

**Published:** 2002-06-01

**Authors:** L. A. Currie, B. A. Benner, J. D. Kessler, D. B. Klinedinst, G. A. Klouda, J. V. Marolf, J. F. Slater, S.A. Wise, H. Cachier, R. Cary, J. C. Chow, J. Watson, E. R. M. Druffel, C. A. Masiello, T. I. Eglinton, A. Pearson, C. M. Reddy, Ö. Gustafsson, J. G. Quinn, P. C. Hartmann, J. I. Hedges, K. M. Prentice, T. W. Kirchstetter, T. Novakow, H. Puxbaum, H. Schmid

**Affiliations:** National Institute of Standards and Technology, Gaithersburg, MD 20899-0001, USA (NIST); Centre des Science du Climat et de l’Environment, Ave de la Terasse, 91 198 Gif sur Yvette, Cedex, France (LSCE); Sunset Laboratories, Inc. 2221 Yew St., Forest Grove, OR 97116, USA (SLI); Energy and Environmental Engineering Center, Desert Research Institute, Reno, NV 89512, USA (DRI); Dept. of Earth System Science, University of California, Irvine, CA 92697, USA (UCI); Dept. of Marine Chemistry and Geochemistry, Woods Hole Oceanographic Institution, Woods Hole, MA 02543, USA (WHOI); Institute of Applied Environmental Research, Stockholm University, 10691 Stockholm, Sweden (SU); Graduate School of Oceanography, Univ. Rhode Island, Narragansett, RI 02882, USA (URI); School of Oceanography, University of Washington, Seattle, WA 98195, USA (UW); Atmospheric Aerosol Research, Lawrence Berkeley National Laboratory, Berkeley, CA 94720, USA (LBNL); Institute for Analytical Chemistry, Vienna University of Technology, Getreidemarkt 9/151, A-1060, Vienna, Austria (TUW)

**Keywords:** ^14^C speciation, elemental carbon, fossil and biomass carbon, international intercomparison, SRM 1649a, total carbon certified value

## Abstract

Because of increased interest in the marine and atmospheric sciences in elemental carbon (EC), or black carbon (BC) or soot carbon (SC), and because of the difficulties in analyzing or even defining this pervasive component of particulate carbon, it has become quite important to have appropriate reference materials for intercomparison and quality control. The NIST “urban dust” Standard Reference Material^®^ SRM 1649a is useful in this respect, in part because it comprises a considerable array of inorganic and organic species, and because it exhibits a large degree of (^14^C) isotopic heterogeneity, with biomass carbon source contributions ranging from about 2 % (essentially fossil aliphatic fraction) to about 32 % (polar fraction).

A primary purpose of this report is to provide documentation for the new isotopic and chemical particulate carbon data for the most recent (31 Jan. 2001) SRM 1649a Certificate of Analysis. Supporting this is a critical review of underlying international intercomparison data and methodologies, provided by 18 teams of analytical experts from 11 institutions. Key results of the intercomparison are: (1) a new, *Certified Value* for total carbon (TC) in SRM 1649a; (2) ^14^C *Reference Values* for total carbon and a number of organic species, including for the first time 8 individual PAHs; and (3) elemental carbon (EC) *Information Values* derived from 13 analytical methods applied to this component. Results for elemental carbon, which comprised a special focus of the intercomparison, were quite diverse, reflecting the confounding of methodological-matrix artifacts, and methods that tended to probe more or less refractory regions of this universal, but ill-defined product of incomplete combustion. Availability of *both* chemical and ^14^C speciation data for SRM 1649a holds great promise for improved analytical insight through comparative analysis (e.g., fossil/biomass partition in EC compared to PAH), and through application of the principle of isotopic mass balance.

## 1. Introduction and Overview

What began as an informal exchange of particulate carbon data for SRM 1649a among just a few cooperating laboratories grew into a proper international intercomparison, the results of which are presented here. The focus of the initial work, as well as the ultimate product, was the characterization of the NIST Urban Dust Standard Reference Material (SRM) 1649a for isotopic and particulate carbon—especially ^14^C and “elemental” and total carbon (EC, TC).

An initial driving force for our efforts was the impending re-certification and re-issue of the original urban dust SRM 1649 as SRM 1649a. The “new” SRM is, in fact, derived from the same batch of urban dust resulting from the massive collection in Washington, DC during 1976–1977 [1]. SRM 1649a, which is a new, repackaged batch of SRM 1649, includes data for new measurands as well as more extensive and more precise data on previously certified measurands [2]. The subsequent chain of events leading to the expanded intercomparison included: (1) a special forum (“Symposium on Black Carbon in the Environment”) at the August 1999 Goldschmidt Conference at Harvard University [3]; (2) cooperative efforts between NIST and SRI International to produce a prototype “filter” reference material from the bulk SRM [4]; and (3) contemporaneous BC intercomparisons of representative ambient and source samples for the atmospheric sciences [5–7] and geosciences [8]. As a result, the initial SRM 1649a particulate carbon intercomparison gained significant breadth of perspective and participation. An added, very important outcome of the Black Carbon Symposium was the birth of the International Steering Committee for Black Carbon (BC) Reference Materials [9]. The BC Steering Committee has expanded the search for and characterization of suitable BC reference materials to meet multidiscliplinary needs.

Black carbon intercomparisons, and consequent nomenclature issues, are not new. Already at the 1980 Conference on “Particulate Carbon: Atmospheric Life Cycle” in Warren, Michigan, the importance of intercomparisons for assessing BC measurement difficulties was represented in the presentation of Cadle and Groblicki [10], and it was noted that these difficulties were “further complicated by inconsistencies, redundancies, and contradictions in nomenclature … [and] unique operational terminology …” with particulate carbon descriptors including “elemental carbon … apparent elemental carbon, soot, dry soot, nonvolatile carbon, nonsoluble carbon, absorbing carbon, residual carbon, and total noncarbonate/nonvolatile carbon” [11]. Following the usage in the Certificate of Analysis for SRM 1649a, we have adopted for this intercomparison the generic term “elemental carbon” (EC) to represent results of methods designed to measure various manifestations of the metaphorical “black carbon spectrum.”

### 1.1 Teams vs Laboratories

A Conventional treatment of interlaboratory comparisons identifies results according to which “laboratory” provided the results in question. Such an identifier is reasonable and convenient when there is 1:1 correspondence between individuals (or organizations) providing the data. In the present intercomparison however, in several cases different individuals from a given laboratory, or even from different laboratories, would “team up” to generate results for a given analyte by a certain method. We have decided therefore to identify providers of specific results as *Teams*. (See Appendix 2.)

## 2. Intercomparison Materials; Measurands

### 2.1 Urban Dust (1649a) Bulk Standard Reference Material

The basic material of this intercomparison is the bulk (powder) urban dust reference material that resulted from a massive atmospheric sampling effort that took place in the Washington, DC Navy Yard during approximately a year’s period, in 1976–1977. Quoting from the SRM 1649a Certificate of Analysis: “While the sample is not intended to be representative of the area in which it was collected, it should generally typify atmospheric particulate matter obtained from an urban area. The particulate material was removed from the baghouse filter bags by a specially designed vacuum cleaner and combined into a single lot. This lot was passed through a 125 μm (120 mesh) sieve to remove bag fibers and other extraneous materials. The sieved material was then thoroughly mixed in a V-blender and bottled.” A very large amount of material was collected, ≈ 20 kg. This has allowed the “Washington” urban dust to be broadly characterized by a number of teams (laboratories), and it guarantees a supply large enough to capitalize on the detailed characterization for a number of years to come.

Among the principal SRM 1649a measurands, we find: polycyclic aromatic hydrocarbons (PAH), polychlorinated biphenyls and chlorinated pesticides, polychlorinated dibenzo-*p*-dioxins and dibenzofurans, a series of 32 inorganic constituents, and special physical and biological characteristics: particle size and mutagenic activity, respectively. Another measurand, having specific relevance to the present intercomparison, is carbonate carbon. Early measurements gave an *information value* of about 0.0012 (mass fraction) of this constituent.^1^ This represents less than 0.7 % of the total carbon (TC); hence for this material, the TC may be taken as the sum of the organic (OC) and elemental carbon (EC) to within 1 %. (This sum is designated by some workers as “total organic carbon” (TOC).) The small relative abundance of carbonate carbon in SRM 1649a has the fortunate consequence of minimizing certain artifacts associated with “thermal” methods of OC/EC analysis, where carbonate carbon can be misconstrued as EC.^2^

Small sample heterogeneity is an issue that must be considered both in the utilization of SRM 1649a as a reference material, and in the interpretation of intercomparison data where sample sizes may differ among methods or among teams. Although the material was “thoroughly mixed in a V-blender” prior to bottling, it does not necessarily follow that it is homogeneous at, e.g., the microgram scale; nor can one make the assumption that heterogeneity is independent of analyte. Given the assumption of randomness, however, the “Ingamells’ constant” approach might be used to extrapolate from larger to smaller sample sizes [13]. Some of the data having relevance to SRM 1649a heterogeneity are as follows: (1) For PAH: the analysis of subsamples ranging from 1 mg to 400 mg showed no significant differences in PAH concentrations, and a limit of 1 % was stated for heterogeneity error for sample sizes of 450 mg for PAHs having certified values. (2) Inorganic constituents were determined on duplicates having sample masses of 100 mg or 250 mg. (3) TC was determined at NIST on 0.3 mg to 9 mg portions without evidence of heterogeneity. (4) TC was determined by Team 10 on 0.3 mg to 1.5 mg portions with no evidence of a trend. (5) Yields of EC (“soot carbon”) obtained by Team 4 on three 25 mg (nominal) portions of the SRM showed ≈ 10 % relative standard deviation (rsd), which is therefore an upper limit for the heterogeneity component. This EC variability, however, is trivial compared to the range of EC/TC results which exceeds a factor of seven. Points 1–3, above, derive from Ref. [2]; points 4 and 5 derive from this intercomparison.

Sample heterogeneity as discussed above refers to the bulk SRM. The prototype filter reference material (RM), to be discussed immediately below, is more problematic. Total amounts were generally small (about 3 mg to 5 mg per filter), and in many cases only a fraction of the filter was subjected to analysis. For example, thermal-optical (TC, EC) methods generally used a few 0.5 cm^2^ to 1.5 cm^2^ punches from 37 mm (diameter) filters. Thus, intrinsic heterogeneity of the bulk SRM is confounded with possible filter mass loading variations.

### 2.2 Prototype Filter Reference Material

Optical and thermal-optical methods of EC analysis depend on transmission or reflectance measurements, and yield results that are expressed in units of μg/cm^2^. Such methods require uniform deposits of the SRM, with known loadings. Also, to avoid optical saturation effects, such loadings must be limited to no more than about 10 μg EC/cm^2^ [14]. As a first step to develop an urban dust filter SRM, Klouda and coworkers prepared a set of prototype quartz filters, having uniform deposits of the bulk SRM 1649a. The prototype RM (“ACG series”) was prepared by depositing the Urban Dust onto pre-weighed 37 mm (diam.) quartz filters, using a special chamber that provided for re-suspension of the bulk SRM in air [4].

Subsequent (unpublished) work by Klouda and colleagues have set some bounds for intra- and inter-filter heterogeneity for chemical elements. For carbon, the thermal (*not* thermal-optical) method of Cachier, Bremond, and Buat-Ménard [15], applied by Team 10 gave TC results of 0.181 g/g for one of the prototype filters, and 0.1760 g/g (*u* = 0.0029, *n* = 4) for the bulk SRM. The relative difference (2.8 %) is not statistically significant. (The certified TC value for the bulk SRM 1649a is 0.1768 g/g (*U* = 0.0019). Note that the symbols *u* and *U* represent standard uncertainties and expanded uncertainties, respectively [16].) The corresponding Team 10 results for EC/TC were 0.385 (filter) and 0.347 (bulk SRM). Note that these results do not reflect intra-filter heterogeneity or average mass loading inaccuracy, since the entire filter (in halves) was used in the analysis, and the total mass loading (mg) was known. Because of difficulties of estimating mass loading (mg SRM/cm^2^) and its variability on the 0.5 cm^2^ to 1.5 cm^2^ scale—needed for the thermal-optical methods—we have chosen to draw intercomparison TC data strictly from measurements on the bulk SRM. Values for EC/TC, however, are derived from both bulk and filter sample measurements. For the former, these are given directly by the ratio of the two concentration estimates; for the prototype filter RM, EC/TC is given by the average for the specific punch areas measured. The (filter EC/TC) values should be resistant to loading inaccuracies and variability, to the extent that EC and TC are similarly distributed.

### 2.3 Carbon Isotopes

This intercomparison is the first such exercise to involve both chemical and ^14^C isotopic speciation of a Standard Reference Material. The importance of the isotopic component of the exercise derives from the fact that the SRM represents a “natural matrix” having both fossil and biomass components. Thus, there are opportunities to investigate relations between chemical (carbonaceous) species and carbon isotopes (^13^C, ^14^C), on the one hand, and to test for isotopic-chemical mass balance, on the other. In the present case, that balance has not yet been achieved, meaning that there are important chemical species and related particulate carbon sources that have not been accounted for. Thus, the intercomparison represents more than an exercise in quality metrology; it has also a fundamental isotopic geochemistry research component.

Speciation of naturally-occurring carbon isotopes (^13^C, ^14^C) adds a new dimension to this intercomparison. Certification of the atmospheric reference material for ^14^C is vital for the control of accelerator mass spectrometry (AMS) measurements of “soot” and particulate carbon in the atmosphere, where the resulting data provides direct quantification of contributing fossil and biomass carbon components of the species measured. When considered in combination with complementary chemical information, the isotopic data results in a level of metrological insight and quality control that would not otherwise be available. A case in point is the multivariate relationship involving chemical and isotopic characterization of the combustion tracers, EC and PAH. A second, vital contribution of the carbon isotopes to the quality of the overall characterization of SRM 1649a particulate carbon, is the assessment of isotopic mass balance—i.e., the justification of the ^14^C (^13^C) composition of the total particulate carbon (TC), in terms of the sum of the concentration-weighted ^14^C values of the major carbonaceous species.^3^

#### 2.3.1 14C Reference Values: Fraction of Modern Carbon, *f*_M_

The basic measure for natural ^14^C is *f*_M_, the fraction of modern carbon. This is defined by reference to the international standard for radiocarbon dating. The *f*_M_ value is derived from the ^14^C/^12^C ratio observed, relative to 0.95 times that of the international (oxalic acid) standard, SRM 4990B. The original basis for the reference state for natural radiocarbon (*f*_M_ = 1) was the need to provide a “zero” for the radiocarbon dating time scale. The objective was to define a reference state (^14^C/^12^C ratio or the equivalent radioactivity concentration) that would reflect the value of the living biosphere, prior to ^14^C dilution from the injection of fossil carbon beginning with the industrial revolution, or the enormous enhancement resulting from atmospheric nuclear testing. The original reference artifact was “1890 wood,” but this was replaced in the mid-20th century by the (then) NBS SRM 4990B (oxalic acid dihydrate), which had a ^14^C radioactivity concentration in AD 1950 that was about 5 % greater than the previous (1890 wood) artifact. This, and its intercalibrated successor (oxalic acid SRM 4990C), has remained the primary standard for natural ^14^C in applications ranging from radiocarbon dating to isotope geochemistry. Although SRM 4990B remains the primary standard for these applications, laboratory measurements have shown the reference value (*f*_M_ = 1) to be equivalent to approximately 13.6 disintegrations min^−1^ g^−1^ carbon (≈ 230 Bq/kg) [18].

For detailed information on the definition of *f*_M_, taking into account adjustment for the ^13^C/^12^C ratio (for precise work) see Stuiver and Polach [19] and Hut [20]. Note that all *f*_M_ values are corrected for ^14^C decay to the mid-date of sampling (1977.0 for SRM 1649a) using the physical half life: 5730 a. Corrections are quite small for the measurements, which were made between 1983 and 1999, with correction factors ranging from 1.00073 to 1.00273. Note that *f*_M_ reflects the ^14^C content compared to the artifactually defined “modern” reference state (based on SRM 4990B). At the time of collection of SRM 1649a (1976–1977) the ^14^C content of the living biosphere was approximately 1.35× Modern as a result of atmospheric nuclear testing [21].

#### 2.3.2 Fossil and Biomass Carbon; Isotopic Heterogeneity

For reference materials derived entirely from fossil or biomass sources, ^14^C speciation data would be of little interest, since all chemical components would be “living” or “dead.” For SRM 1649a, however, where only about 40 % of the carbon, on average, is biogenic, there is a wealth of information contained in the varying contributions of fossil and biomass sources to different chemical species, as well as the aforementioned opportunity to use isotopic mass balance for an extra measure of quality control.

As indicated in Sec. 2.3.1, reference values for ^14^C abundances in the chemical species are defined in terms of *f*_M_, the fraction of modern carbon. These are the values that comprise the outcome of the intercomparison, and the values that appear on the Certificate of Analysis of the urban dust standard reference material. Fossil and biomass carbon fractions, which have special importance for source apportionment, may be derived from the *f*_M_ values, by taking into account the time-dependent ^14^C content of the living biosphere. For the reference date (average sampling date) for SRM 1649a (1977.0), the ^14^C/^12^C ratio of the living biosphere was enhanced by a factor of 1.35 as a result of excess ^14^C produced by atmospheric nuclear testing. The excess reached a peak in the mid-1960s with more than a doubling of ^14^CO_2_ in the northern troposphere. Since the cessation of nuclear testing in the atmosphere, biogeochemical relaxation has been manifest as the “biomass ^14^C decay curve” that links the tail of the nuclear testing input function with the year of biomass growth.

**Fig. 1:**
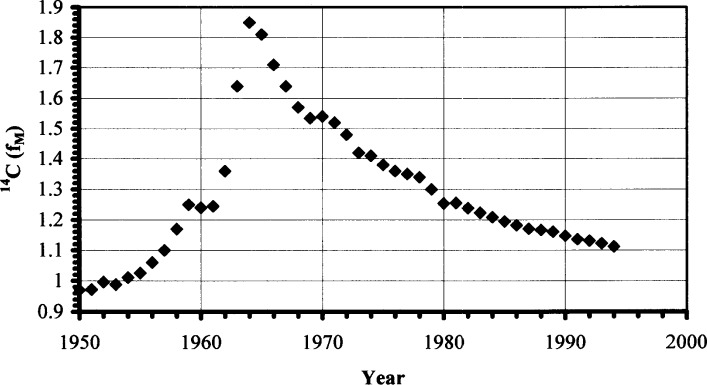
*Biospheric ^14^C enhancement from atmospheric nuclear testing*. The plot shows the time record of ^14^C in the living biosphere, resulting from atmospheric nuclear tests during the 1960s and early 1970s. The ^14^C content of northern hemisphere biomass carbon was approximately doubled in 1963. Since the cessation of atmospheric tests, geophysical relaxation of the excess ^14^C has resulted in a gradual “decay” now approaching the natural, cosmic ray production asymptote. (*f*_M_ relative standard uncertainties are typically less than 0.5 % [21].) At the time that SRM 1649a was collected from the atmosphere, the biomass ^14^C was enhanced by a factor of about 1.35.

The complete (northern hemisphere) input function is shown in Fig. 1 [21]. The post-treaty (atmospheric nuclear test ban) “decay curve” shows that biomass carbon was 1.35 times Modern in 1977. Thus, for SRM 1649a, the fraction of biomass carbon, denoted “contemporary carbon” (*f*_C_) [22], equals *f*_M_/1.35. The fraction of fossil carbon is then 1 − *f*_C_. For source apportionment studies, caution must be exercised in the interpretation of *f*_C_, in cases where the biomass material is not fully contemporary. This occurs when there are biological compartments having different time constants, and especially in biomass burning studies where the wood burned may span several years of growth. In that case, the atmospheric ^14^C input function must be appropriately integrated [23].

## 3. Measurement Techniques Applied

Three classes of measurements are represented among the intercomparison results: Total Carbon (TC); Elemental Carbon (expressed as the ratio, EC/TC); and ^14^C speciation. TC and ^14^C data were taken from measurements performed directly on the bulk SRM (powder) only, to avoid possible uncertainties in loading and uniformity of the prototype filter RM. EC/TC ratios, on the other hand, were drawn from both intercomparison materials (bulk SRM 1649a and prototype ACG filter deposits of the resuspended reference material.) In a few selected cases that will be discussed in Sec. 4 of this document, data for TC and EC/TC were obtained (by the same method) on both the bulk SRM and prototype filters, permitting comparability tests for the two materials.

TC ideally would refer to total non-carbonate carbon, thus comprising both organic carbon (OC) and elemental carbon (EC), and sometimes known as total organic carbon (TOC). We do not favor the use of TOC for this purpose, however, because of ambiguity with TC as the sum of organic and elemental carbon components. Regarding carbonate, however, earlier measurements on large (0.7 g to 1.0 g) samples of the bulk SRM showed that carbonate carbon was but 0.66 % of TC. This was fortunate, in that not all participating teams applied decarbonation pretreatment.

TC results on the bulk SRM, submitted by seven teams, were of sufficient quality to generate a certified value for TC. In all cases combustion in oxygen (or dilute mixtures of O_2_ in He) was employed to generate the CO_2_ for quantification. Differences among the TC methods are treated below in Sec. 3.2.

^14^C data were obtained by low-level decay counting (llc) or accelerator mass spectrometry (AMS). Speciation was accomplished by applying appropriate chemical isolation procedures to the bulk SRM prior to llc or AMS. These are presented in Sec. 3.4.

EC/TC ratios, for the bulk SRM and/or the prototype filter RM, resulted from the application of three primary approaches for the determination of elemental carbon: optical, thermal, and chemical. Optical transmission, using an appropriate attenuation coefficient, yields a direct measure of EC, given certain assumptions. Thermal, or “dry oxidation” techniques serve to discriminate OC from EC on the basis of the relative resistance of the latter to volatilization and reaction with oxygen. Chemical or “wet oxidation” techniques rely on the resistance of EC to strong chemical oxidizing agents in solution, such as oxidizing acids and hydrogen peroxide.

Hybrid techniques abound, in particular thermal-optical and thermal-chemical techniques. The former class utilizes EC transmission or reflectance to monitor the progress of thermal oxidation, and in many cases to correct for pyrolytic formation of artifact EC (“charring”). Thermal-chemical techniques approach the problem by prevention, where chemical pretreatments are designed to remove species subject to high temperature pyrolysis. Two methods [TOK, Ch(Cr)K—terms to be defined below] explicitly incorporate kinetic analysis, where the asymptote (intercept) is estimated when fitting a sum of exponentials.

Brief descriptions and citations follow, for the principal techniques utilized for TC, EC/TC, and ^14^C, respectively.

### 3.1 Small Samples and Isotopic Assay: Some Special Considerations

The need for accurate EC metrology spans a number of disciplines, from atmospheric aerosol science to marine geochemistry to isotopic biogeochemistry. All of these disciplines have needs for EC reference materials and EC reference methods [9]; and many have developed methods specially suited for their characteristic samples. Since the present SRM intercomparison was designed to address some of these multidisciplinary needs, participants and methodology were drawn from their respective communities.

New challenges resulting from this broadened scope were: (1) the application of chemical oxidation techniques that were developed both for large samples (e.g, gram quantities of marine sediment) and for small samples (e.g., mg quantities of atmospheric aerosol); and (2) samples that could be readily treated in bulk vs those requiring special “micro” techniques. The latter include *in situ* thermal optical reaction monitoring on small quartz filters, and isotopic speciation involving quantitative isolation of 10 μg to 100 μg of selected species for ^13^C (IRMS) or ^14^C (AMS) assay. As a result, some of the following methods for EC and for ^14^C speciation demanded some rather significant adaptations of “macro-” techniques to allow us to perform “chemistry on a filter” and to minimize both losses and blanks. Especially difficult are the problems of constraining EC particle-loss to less than 1 μg while performing chemical oxidation or solvent extraction on mg amounts of the SRM, or processing small samples for ^14^C speciation where contamination of both fossil and contemporary carbon must each be kept below the microgram level. The special approach to small samples is illustrated by one of the more complex, wet and dry oxidation, multi-reagent procedures—“Ch(N1)T”—which has been treated in extra detail below in Sec. 3.3.3.

### 3.2 Total Carbon Methods

#### A. Thermal optical transmission/combustion [24]

In an oxygen-free helium atmosphere, the sample (≈ 1 mg) was heated in four increasing steps to about 820 °C to remove organic carbon. Organic compounds that are pyrolytically converted to elemental carbon were continuously monitored by measuring the transmission of diode laser light through the filter. As organic compounds are volatilized, they are immediately oxidized to CO_2_ using a plug of MnO_2_ at 860 °C; the CO_2_ is reduced to methane over Ni on firebrick in the presence of H_2_, and measured using a flame ionization detector (FID). After cooling the sample to 525 °C, a 2 % (or 5 %) O_2_/He mixture was introduced and the temperature increased in two steps to about 860 °C. Total carbon is derived from the total integrated signal, and calibration with substances of known stoichiometry. [Teams 5, 8] (Method Code for Table 1: Combust(TOT).)

#### B. Combustion-GC-TCD [25]

Samples were weighed into Al boats, combusted to CO_2_ at 900 °C in an atmosphere of O_2_, purified by gas chromatography (GC), quantified with GC using a thermal conductivity detector (TCD). [Team 18] (Method Code for Table 1: Combust(GCTCD).)

#### C. Combustion-NDIR (NIST) [4]

The weighed sample was placed in a ceramic crucible which was then purged with O_2_ while inductively heating the crucible. The CO and CO_2_ produced were measured using a non-dispersive infrared (NDIR) detector. [Team 9] (Method Code for Table 1: Combust(NDIR).)

#### D. Flash combustion (elemental analyzer) [26]

Samples were placed in Ag capsules, subjected to repeated *in situ* mild microacidification (1 *M* HCl) to remove carbonates, and then fully oxidized and quantified by flash combustion/gas chromatographic analysis using a commercial carbon-hydrogen-nitrogen (CHN) analyzer. This process served also as the final carbon quantification step for the T375 method described below for the determination of elemental carbon. [Teams 3, 6] (Method Code for Table 1: Combust(CHN).)

#### E. Two-step thermal combustion) [15]

Samples are first decarbonated with HCl vapor (excess removed with NaOH pellets), and then flash heated in a stream of oxygen at 340 °C for 2 h to remove OC. Final combustion of the remaining EC component takes place at 1100 °C. CO_2_ from both steps is quantified by coulometric titration. [Team 10] (Method Code for Table 1: Combust(2step).)

### 3.3 Elemental Carbon Methods

Note that abbreviated names, suggestive of the respective methods, are given at the beginning of each method description. These will be used in the following section and in Table 2 to efficiently link results with specific methods.

#### 3.3.1 Optical Attenuation

##### AETH (Optical transmission, “aethalometry”) [27]

Bulk SRM (≈ 1 mg) was loaded on a 25 mm diameter quartz fiber filter, and the attenuation of a visible light beam was compared to that of a blank filter. EC loading was calculated from the observed attenuation using an EC attenuation coefficient of 19 m^2^/g, as recommended by the manufacturer of the Aethalometer for aerosol deposited on quartz fiber filters. (It should be noted that a range of values for the attenuation coefficient has been reported in the literature: from about 5 m^2^/g in remote regions to about 20 m^2^/g in urban locations [28, 29].) [Team 1]

#### 3.3.2 Selective Thermal (“Dry”) Oxidation and Thermal-Optical Hybrids

##### T375 (Thermal oxidation) [26, 30]

Samples were weighed into Ag capsules, carbonates removed by repeated mild *in situ* acidification (1 *M* HCl), followed by volatilization and thermal oxidation at 375 °C for 24 h in air with continuous supply to ensure excess oxygen. The isolated EC residue was quantified by flash combustion/gas chromatographic analysis using commercial CHN analyzers. The more recent publication [30] reports on an extensive optimization and interference study, and introduces the acronym CTO for this “chemo-thermal oxidation method.” [Teams 2–4, 6]. Adaptations: Team 3 used a tube furnace with continuous air flow, whereas Teams 2, 4, and 6 used muffle furnaces; Team 4 calculated EC from the CO_2_ yield from closed tube combustion for ^14^C (EC) analysis; Team 6 used a 12 h oxidation step at 375 °C.

##### T340 (Two-step thermal oxidation) [15]

Samples are first decarbonated with HCl vapor (excess removed with NaOH pellets), and then flash heated in a stream of oxygen at 340 °C for 2 h to remove OC. Final combustion of the remaining EC component takes place at 1100 °C, and the resulting CO_2_ is quantified by coulometric titration. [Team 10; adaptation of the method by Team 7 omitted the decarbonation step, and used an elemental (CHN) analyzer for the final EC quantification.]

##### T500 (Evolved gas analysis, EGA) [14]

A thermal oxidation method in which the sample combustion takes place in a stream of oxygen over the temperature range: 50 °C to 800 °C, with EC defined by highest temperature peak, which is generally centered at ≈ 500 °C. To minimize the effect of OC on the low temperature side of the peak, the full peak area is taken as twice that of the high temperature side. OC artifacts are minimized by pretreatment (solvent extraction: acetone, hexane, methanol); and mechanical EC loss during extraction is monitored optically by laser transmission. Reliable transmission monitoring requires that sample loading not exceed 10 μg EC per cm^2^. [Team 14]

##### TOT (Thermal optical transmission) [24]

In an oxygen-free helium atmosphere, the sample (≈ 1 mg) on a 1 cm^2^ to 1.5 cm^2^ quartz fiber filter punch undergoes stepwise heating from about 60 °C to 900 °C to volatilize and/or decompose organic carbon. Char formation from the pyrolysis of organic matter is monitored continuously by measuring the transmission of a 670 nm diode laser beam through the filter. As organic compounds are volatilized, they are immediately oxidized to CO_2_ using a plug of MnO_2_ at 860 °C, reduced to methane over Ni on firebrick in the presence of H_2_, and measured using a flame ionization detector (FID). After cooling the sample to 525 °C, a 5 % O_2_/He mixture is introduced and the temperature increased stepwise to about 900 °C. Based on the FID response and laser-transmission data, the amounts of organic, elemental, and pyrolytic carbon are then calculated for the sample. [Teams 5, 8, 18]^4^

##### TOR (Thermal optical reflectance) [6]

A 0.5 cm^2^ punch of the sample (quartz) filter was subjected to a stepped temperature program in a flow of helium (120 °C, 250 °C, 450 °C, and 550 °C), using MnO_2_ at about 910 °C to convert volatilized OC to CO_2_. This was followed by oxidation of residual carbon (EC) in a flow of O_2_ (2 % in He) with temperature steps at 550 °C, 700 °C, and 800 °C. The switch (He to O_2_) took place at the “split time for pyrolysis correction,” determined by monitoring reflectance with a diode laser. CO_2_ was determined with an FID detector after being reduced to CH_4_ with H_2_ (Ni catalyst). [Team 11]

##### TLT (TOT, with linear temperature program) [31, 32]

The bulk SRM spread on a quartz fiber filter as well as samples of the prototype filter RM (ACG), were processed in a stream of O_2_ from laboratory temperature to 800 °C with a linear temperature ramp of 20 °C per min, with CO_2_ quantification by NDIR. Transmission measurement with a laser diode was used to determine the split time for pyrolysis correction. [Team 12]

##### TOK (Thermal kinetic oxidation/intercept-EC) [33, 34]

EC is defined as the refractory (intercept) component that survives isothermal oxidation at 560 °C in a stream of He(5 % O_2_). The intercept-EC is estimated by fitting a five-parameter model (two exponentials + intercept) to the residual carbon rate function. Loss of more labile EC is monitored during oxidation by transmission of a diode laser beam. [Team 5]

#### 3.3.3 Selective Chemical (“Wet”) Oxidation and Thermal-Chemical Hybrids

##### Ch(N1)T (HNO_3_-thermal oxidation)—an archetype for “micro” chemical and isotopic speciation

The Ch(N1)T procedure was adapted from a hybrid wet-dry oxidation procedure developed for the analysis of “soot carbon” in bulk soil samples [35]. The miniaturized version was developed at NIST for this intercomparison and for subsequent application in atmospheric and cryospheric isotopic chemistry. It represents perhaps the first time that wet chemical oxidation has been applied to the isolation of sub-mg amounts of EC, with subsequent ^14^C speciation by small sample AMS [36]. Its key attributes are “wet chemistry on a filter” and “2-stage thermal oxidation in a combustion tube.” As indicated in Sec. 3.1 the description given here has been expanded to illustrate the special considerations needed to adapt a “macro” procedure for EC to “micro” assay of EC and ^14^C-EC in small atmospheric samples.

The basic process, which includes acid-base pretreatment followed by 2-step thermal oxidation, was adapted to operate on the bulk SRM, distributed on a 25 mm quartz filter using a vortex mixing-filtration procedure. For mg sized samples, minimization of reagent amounts and the performance of reactions on quartz filters rather than in test tubes, are important for the reduction of both particle loss and blanks. NaOH assists in the hydrolysis of natural biopolymers, which tend otherwise to char in thermal processing; and concentrated (laboratory temperature) HNO_3_ serves as the “wet chemical” oxidizing agent. Laboratory temperature HNO_3_ is used to minimize attack on EC [10]. The second stage, a two-step thermal process adapted from Cachier, et al. [15], is again miniaturized to suit small, closed tube (CT) combustion procedures, essential for quantitative recovery of *both* the OC- and the EC-derived CO_2_ for the preparation of small AMS targets for ^14^C assay [37].

The first step is to achieve the efficient transfer of milligram and submilligram amounts of particulate carbon—from the SRM, or from ice core meltwater—to a small quartz filter. For the present intercomparison, the goal was to transfer 1 mg to 1.5 mg of the bulk SRM 1649a to 25 mm quartz filters, which had been prefired for 3 h at 900 °C, to minimize the filter blank. Transfer was accomplished by dispersing the material in 50 mL of prefiltered distilled water and vortex mixing for 2 min, followed by vacuum filtration, with repeated passage of the filtrate through the filter. This was followed by drying in a desiccator for 24 h to 36 h.

The first chemical pretreatment stage is designed to eliminate inorganic carbon, and polymeric organics that are apt to form artifact EC (char) in thermal processes. In the “micro” adaptation, gravity feed was used, with 5 mL each of the following reagents added in sequence: 1 *M* NaOH (twice), 70 % (mass fraction) HNO_3_ (once), 1 *M* NaOH (thrice), 1 % (mass fraction) HCl (once), and prefiltered distilled H_2_O (twice). Following a final rinse with 100 mL of distilled water with vacuum filtration, the sample was oven dried at 105 °C for 2 h.

The second, thermal oxidation stage utilized two-step closed tube combustion, where 380 mbar (38.5 kPa) of high purity O_2_ was added to an evacuated quartz tube containing the sample together with CuO and Ag wire. The first thermal step eliminated residual OC by heating the sealed quartz tube at 340 °C for 2 hours, followed by evacuation of the evolved gases^5^; in the second step the residual EC was oxidized by closed tube combustion at 950 °C. After cryogenic purification, the CO_2_ was quantified by calibrated volume manometry [39]. [Team 7] For the smallest samples (<25 μg C), accelerator targets were prepared using the NIST-Woods Hole “dilution-AMS” procedure [36]. A unique problem that came with vigorous, multistage chemical and thermal processing was the introduction of trace impurities that: (1) interfered with the cryogenic purification of CO_2_, leading to erroneous volumetric dilution factors, and (2) sometimes led to poor performing accelerator targets because of interference in the catalytic reduction of CO_2_ in the final step of “graphitic” AMS target fabrication [36].

##### Ch(N2)T (HNO_3_-thermal oxidation) [39, 40]

The Ch(N2)T two-stage, chemical-thermal oxidation procedure for mg sized samples is similar to Ch(N1)T, except that pretreatment steps with NaOH were omitted in an effort to minimize the chemical blank and losses for small samples. “Wet-chemical” oxidation took place with laboratory temperature 70 % (mass fraction) HNO_3_; and thermal, two-step closed tube oxidation took place at 340 °C (2 h) and 950 °C, with O_2_ and CuO as the respective oxidizing agents. [Teams 1, 7]

##### Ch(N3) (Chemical oxidation, hot HNO_3_) [25, 41]

This method was applied to large samples such as aerosol deposits on 10 cm^2^ to 20 cm^2^ portions of “hivol” quartz filters. For SRM 1649, about 1 g of the bulk material was placed in a 100 mL beaker and treated with 25 mL boiling 70 % (mass fraction) HNO_3_ for 20 min. Next, 35 mL of 6 *M* HNO_3_ was added and the mixture was allowed to stand overnight. This was followed by centrifugation, and rinsing of the residue four times with distilled water. The final residual carbon (EC) was quantified by the inductive furnace combustion-NDIR technique. [Team 9]

##### Ch(Cr)K (Chemical oxidation, dichromate/residue, “Wolbach”) [42, 43]

Wet oxidization with 0.25 *M* Cr_2_O_7_^=^ in 2 *M* H_2_SO_4_ at 23 °C was performed on the bulk SRM for periods up to 406 h. The 406 h residual carbon was taken as EC. Kinetic analysis showed that the oxidation process could be represented as a sum of two exponential components (half lives: 0.85 h ± 0.31 h, and 1003 h ± 430 h). [Team 13]

##### Ch(N4) (HNO_3_, “Verardo”) [44]

A 5 mg portion of the bulk SRM was placed in an Al boat, and treated with 300 μL of hot, concentrated HNO_3_, in 30 μL increments. After oven drying overnight at 60 °C, the residual carbon was quantified with an elemental (CHN) analyzer. It was observed that, due to the lack of rinsing, the results could be high if there is a partially oxidized carbonaceous residue. [Team 13]

### 3.4 ^14^C Speciation

#### 3.4.1 Total Carbon

##### Combustion-Manometry [45]

Samples were combusted to CO_2_ in a quartz furnace filled with 101 kPa O_2_. Downstream from the combustion furnace is a series of three furnaces: (1) Pt gauze at 900 °C, (2) CuO at 800 °C, and (3) Ag wool at 400 °C to assure complete combustion and to purify the CO_2_ of sulfur and halogen containing impurities. The sample gas stream is then reduced to less than 13 kPa to prevent the condensation of liquid O_2_ by controlling the gas flow through the system using a throttle valve and a vacuum pump. Before the vacuum pump, the sample CO_2_ is cryogenically trapped at liquid N_2_ temperature (−196 °C) in a series of spiral glass traps. The resulting CO_2_ is cryogenically separated from other gaseous combustion products by distillation from −78 °C and quantified using manometry in a calibrated volume. Low-level ^14^C decay counting was performed on the CO_2_ using a miniature gas proportional counter at NIST [46]. [Team 16]

##### H_3_PO_4_-Combustion-Manometry [47]

A subsample of SRM 1649a, Ag foil (prefired at 550 °C), and CuO wire (prefired at 850 °C) were added to a quartz tube. Approximately 5 mL of 3 % H_3_PO_4_ (mass fraction) was added to the tube to remove any inorganic carbon. The quartz tube was then attached to a vacuum line, evacuated to a pressure of less than 5 Pa, sealed, and combusted to CO_2_ at 850 °C for 4 h. The CO_2_ was reduced to graphite over Co catalyst at 850 °C in the presence of H_2_. Accelerator mass spectrometry ^14^C measurements were performed at Lawrence Livermore National Laboratory. [Team 13]

##### Combustion-GC-CHN [48]

Samples were placed in Sn boats and fully oxidized and quantified by flash combustion/gas chromatographic analysis using a commercial CHN-analyzer, by essentially the same procedure used for TC quantification by Combustion-GC-TCD. The purified CO_2_ was then trapped at −196 °C and transferred to the Univ. Arizona for preparation of graphite targets and ^14^C AMS [49]. [Team 17]

#### 3.4.2 Elemental Carbon

##### Thermal oxidation/residue [50]

^14^C is measured in the residual carbon after thermal oxidation at 375 °C for 24 h (to remove labile organic carbon) and acidification (to remove inorganic carbonates). The residual carbon is placed in a quartz tube containing copper oxide and elemental silver, and combusted at 850 °C for 5 h. The ^14^C content of the resulting CO_2_ was measured by accelerator mass spectrometry at Woods Hole. [Team 4]

##### Chemical oxidation/residue [42]

Wet oxidization with 0.25 *M* Cr_2_O_7_^=^ in 2 *M* H_2_SO_4_ at 23 °C was performed for periods up to 406 h. The ^14^C AMS result is given for the residual carbon for the longest (406 h) reaction period. Because of the gentler oxidation treatment (chemical vs thermal), the ^14^C must reflect somewhat less refractory manifestations of elemental carbon. [Team 13]

##### Thermal kinetic oxidation/intercept-^14^C [33, 34]

EC is defined as the refractory (intercept) component that survives isothermal oxidation at 560 °C in a stream of He (5 % O_2_ [*v*]). This intercept-EC is estimated by fitting a five-parameter model (two exponentials + intercept) to the residual carbon rate function. Intercept-^14^C is estimated as the corresponding end point of a series of three intermediate samples taken for ^14^C AMS. [Team 5]

#### 3.4.3 Selected Organic Fractions

##### Aromatic Carbon

###### Soxhlet extraction/LC isolation of the aromatic fraction [51]

Samples were Soxhlet extracted for 24 h with dichloromethane. The extract was concentrated to a small volume under a stream of N_2_. The concentrated extract was placed on a silica solid phase extraction (SPE) cartridge and eluted with 10 % (*w*) dichloromethane in pentane. The aromatic fraction was isolated using normal phase liquid chromatography on an aminopropylsilane column. This fraction was concentrated and a 1 mL aliquot transferred to a quartz tube and evaporated to dryness. CuO was added to the tube which was then attached to a vacuum line, evacuated, sealed, and the contents combusted to CO_2_. Low-level ^14^C decay counting was performed on the sample CO_2_ using a miniature gas proportional counter at NIST [46]. [Team 16]

##### Polar, Aliphatic, and Aromatic/PAH Carbon

###### Soxhlet Extraction/LC/PCGC/AMS [51, 52]

Extraction followed by LC was used for isolating Polar, Aromatic, and Aliphatic fractions for ^14^C analysis. The aromatic fraction was specially purified using silica gel flash chromatography and LC PAH ring size fractionation, prior to separation and collection of individual PAH using an automated preparative capillary GC (PCGC) system [50]. The individual PAH fractions were then subjected to closed-tube combustion and ^14^C determined by AMS [53, 54]. [Teams 4, 15]

## 4. Results and Discussion

Intercomparison results are presented separately for the three measurand classes (TC, EC/TC, and ^14^C speciation) for the methods described above, and the coded teams. A reminder regarding the Team codes: The numerical Team codes do not necessarily convey different institutions; but rather they indicate which operator or group of collaborators were responsible for the individual results. In one case, for example, where the same (nominal) method was employed by two different operators in the same institution, it was desirable to distinguish the two sets of results by giving them different Team codes. (See Appendix 2.)

### 4.1 Total Carbon

Total carbon (TC) results were drawn strictly from intercomparison data derived from bulk SRM measurements. In the two cases where method A—Combust(-TOT)—was used, the bulk material was carefully spread and weighed on the quartz filter that was inserted into the combustion zone. Results, estimates and standard uncertainties (u), for the seven qualifying data are given in Table 1. (See Sec. 3.2 for descriptions of the five TC methods, together with the abbreviated method codes used in the table.) Since the results are all consistent within the stated uncertainties, the Certified Value for TC was taken as the weighted mean, 0.1768 ± 0.0008 (g/g) where the standard uncertainty is based on the absolute weights.^6^

Two considerations were important in deriving the TC certified value: carbonate carbon, and heterogeneity. As mentioned previously, carbonate removal was applied as a pretreatment in some cases, but not in all. Team 10 did it both ways; the result from that pretreatment is reported in Table 1. Fortunately, carbonate carbon is negligible. The information value given in the Certificate of Analysis for SRM 1649a is but 0.66 % of the TC value; the independent estimate (and standard uncertainty) based on the difference between the two results from team 10, is − (1.4 ± 2.1) % of the TC value.

Heterogeneity, as reflected by varying TC content with mass of the SRM taken for analysis was investigated by Klouda for small samples of the bulk SRM [25]. No significant dependence on sample size was seen over the range of about (0.3 to 9) mg of the bulk SRM. An approximate bound for TC heterogeneity over this range is 1.7 % relative. Heterogeneity was tested separately for PAHs. On p. 3 of the new certificate of analysis, issued 31 January 2001, it is stated that “analyses of subsamples of 1 mg to 400 mg show no significant differences in the PAH concentrations, and a sample size of approximately 450 mg will contribute less than 1 % error due to sample homogeneity (sic) for the PAHs for which certified values are provided.” [2]. Additional relevant information for the newly certified lot of SRM 1649a includes the unit size (2.5 g), and the expiration date of the certification: 30 June 2007. (See the following section for heterogeneity data for EC/TC.)

### 4.2 Elemental Carbon^7^

In a special sense, elemental carbon (EC) lies at the heart of this intercomparison. It has central importance in linking particle emissions from natural and anthropogenic combustion with potentially deleterious effects of “Black Carbon” (BC) on visibility, health, and climate. Yet, BC is neither a unique substance, nor an entity for which consensus nomenclature or measurement data obtain. A key objective of this intercomparison has been to examine patterns of results from the perspective of the diverse chemical and physical characteristics of a broad array of BC measurement techniques. No method or result can in principle be judged “correct” (or incorrect), but it is hoped that insight may be gained on the utility of different techniques to probe different aspects of this complex material.^8^ To minimize nomenclature confusion, this remarkable state of matter is referred to as elemental carbon (EC) in the SRM 1649a certificate of analysis as well as in this manuscript.

Experimental data for SRM 1649a EC, expressed as the dimensionless ratio EC/TC, are presented in Table 2, along with teams and methods from Sec. 3.3. Note that data based on optical or thermal optical measurements of the prototype (ACG) reference material are shown in italics, to distinguish them from data for the bulk Standard Reference Material. When determined with a small punch from the prototype filter RM, these processes give results in μg EC/cm^2^ and, except for aethalometry (AETH), μg TC/cm^2^. The ratio (EC/TC) for the particular punch follows directly without the need to know the mass loading (mg/cm^2^). To the extent that the ratio is independent of variations in mass loading, it should be more robust than either of the elemental or total carbon values.

#### 4.2.1 Descriptive Overview of the Data

**Fig. 2:**
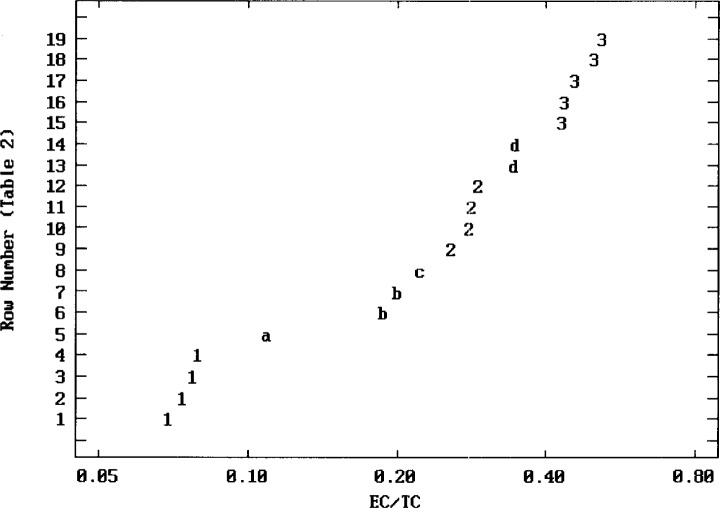
*Empirical cluster display of the EC/TC data*. Ordered results (≈ 7 % to 50 % EC) from the 19 method-team combinations of Table 2 are displayed on a log axis (for variance stabilization), with clusters identified by the quantitative gap cluster algorithm [56]. Descriptive statistics [medians (quartiles)] for the three primary clusters (*n* > 2) are: 1) 0.075 (0.071, 0.078) 2) 0.28 (0.27, 0.29) 3) 0.46 (0.44, 0.50). The remaining four secondary clusters are comprised of two singletons (a,c) and two pairs (b,d). Smaller values of EC/TC may be interpreted as EC loss (artifact) and/or more refractory EC (“soot”). Larger values may be interpreted as OC captured in the EC fraction (artifact) and/or more labile EC (“char”). Result-specific physical-chemical bases for such trends are discussed in the text.

Complementing the “view” of the data given in tabular form, it is useful to provide a descriptive graphical summary, relatively free from assumptions, such as normality *or even unimodality*. Inspired by the “black carbon spectrum” metaphor, with different analytical methods probing different parts of that spectrum, it has been interesting to apply a multi-modal method of graphical data summarization, based on the “quantitative gap” approach for representing complex intercomparison data in chemical metrology [56]. The resulting visual summarization of the entire EC/TC dataset is given in Fig. 2.

Based on the modality (number of major, *n* > 2, clusters) displayed, it is useful to further summarize the data with robust boxplots or the corresponding exploratory statistics for each such cluster. Taking the latter course, we give medians and inter-quartile ranges for clusters 1, 2, 3. Note that the full dataset spans a range of 0.069, 0.520 (factor of 7.5.)

**Table:** 

Cluster (*n*, membership)	Median (EC/TC)	Quartiles (lower, upper)
1 (*n* = 4)	0.075	0.071, 0.078
2 (*n* = 4)	0.28	0.27, 0.29
3 (*n* = 5)	0.46	0.44, 0.50

This exploratory summarization of the intercomparison results must not be over-interpreted. It is offered, as with classical cluster analysis, to give a visual grasp of the data structure, and perhaps suggest regions of the data that merit further exploration in terms of their physical and chemical implications.^9^ Consideration of such implications follows in Sec. 4.2.2.

#### 4.2.2 Methodological Contrasts and Artifacts

##### Chemical vs thermal oxidation

One of the more striking contrasts between methods is the use of (wet) chemical vs (dry) thermal oxidation. We note that thermal method results (codes including ‘T’) cover the full range of EC/TC values, whereas results from pure chemical oxidation methods (codes excluding ‘T’) are restricted to the upper regions (EC/TC ≥0.29). This is consistent with the greater resistance of polymeric hydrocarbon structures to chemical as opposed to thermal oxidation, and it suggests that vigorous thermal oxidation may be required for isolation of the most resistant soot component of elemental carbon. For both, however, there are matters of kinetics, to be considered below.

##### Sample heterogeneity and particle loss

For EC/TC analysis there are two sample heterogeneity issues to consider: variations with sample size (mass of the bulk SRM taken for analysis), and variations in loading within and between samples loaded on filters—primarily for analysis by optical or thermal optical methods. While definitive information on these matters is not yet available, some very useful preliminary information is contained in some of the participants’ results.

First, regarding bulk sample heterogeneity and recovery: Participant (Team) number 7, performed a series of measurements with method Ch(N2)T covering the range of 0.9 mg to 13.6 mg bulk SRM. The results indicate that reasonably reproducible values [≈ (3 to 4) % rsd] obtain for 2 mg and above, whereas greater scatter and decreased EC was found with smaller sample sizes.

This brings up the critical matter of particle loss that may accompany chemical processing of very small samples (See also Sec. 3.1). It should be noted that in the limited study variations due to particle loss and EC heterogeneity are somewhat confounded. Two examples of particle loss arose in the intercomparison. The first is the decreased recovery of EC noted above, where apparent EC content dropped by about 15 % (relative) for 1 mg samples of bulk SRM. The second occurred when Team 14 attempted to apply organic solvents to ACG prototype filter samples using method T500_EGA. Again, because of particle loss (from the filter), this extraction step could not be performed reliably.

Ironically, the probable impact on the EC/TC results given in Table 2 is reversed for the two methods. That is, for method Ch(N2)T particle loss would lead to EC data that are biased low; whereas inability to apply OC solvent extraction to ACG filters without particle loss with method T500_EGA could lead to positive bias because of OC masquerading as EC in the high temperature combustion peak. Note that the pre-extraction upper bound for EC/TC, 0.50, is given in Table 2, row 18. The lower bound, following extraction with possible particle loss is 0.40.

##### Filter loading and optical data

Optical and optical-hybrid methods applied to the SRM 1649a, or to the prototype filter RM, depend on valid and representative measurements of transmission (or reflectance). Four artifacts are of concern. First, for the purely optical method AETH, the surface density (EC/cm^2^) is derived from transmission (sample compared to blank filter) and an assumed attenuation coefficient. For aerosol distributed on quartz filters the manufacturer of the aethalometer recommends a value of 19 m^2^/g for EC (used for the AETH result in Table 2). Investigations of aerosol samples from diverse regions, however, indicate that there can be wide variations in this coefficient, roughly (5 to 20) m^2^/g [28]. Taken at face value, this range would suggest that the AETH result in Table 2 might be considered a lower limit for EC/TC. Second, non-linearity in transmission sets in at about 10 μg EC/cm^2^, rendering measurements on heavily loaded filters unreliable. This was a problem with the attempt to monitor EC loss optically following solvent extraction of ACG filters by Team 14, where EC loading was found to be 30 μg/cm^2^ or more. Third, intra- and inter-filter loading heterogeneity add to the imprecision of absolute (μg EC/cm^2^) measurements. This problem may be exacerbated for concentration data (μg EC/mg SRM), as this requires the deposit to be uniformly distributed over a known area. Once again, there can be confounding if deposits are both variable and near saturation (optically). A fourth potential problem, for optical methods that rely on “return to baseline” transmission to define a split point for thermal pyrolysis correction, could be differences in the effective attenuation coefficient for the initial EC and the absorbing pyrolysate. (It is not known whether such a possibility has been effectively ruled out for thermal-optical techniques.) Clearly, any OC that combusts beyond the presumed split point will mimic EC and lead to an inflated value of the latter.

##### Premature oxidation of EC

The complementary problem is EC loss prior to the presumed EC combustion window. This can lead to negative EC bias, especially for non-optical thermal techniques and for “unusual” sample matrices that can catalyze EC oxidation at lower temperatures. Oxidizing metal oxides are a case in point [6, 25], as are non-metallic and organic matrix components that can release oxygen during the volatilization cycle. Early oxidation of “Dark OC,” such as melanoidins [9], represents another artifact that may mimic premature EC oxidation.

##### Comparative EC/TC ratios for the bulk SRM and the prototype filter

EC/TC values in Table 2 are disjoint in the sense that many derive from measurements of the bulk SRM 1649a whereas some (italicized) are based on measurements of the prototype filter RM which was prepared by resuspension of the bulk SRM. Some insight regarding EC/TC intercomparability for the two materials has been given by Teams 8, 10, and 12. Team 8, using TOT, obtained the tabulated value 0.258 ± 0.014 (*n* = 3) for the bulk SRM (spread on a filter), compared to 0.264 ± 0.007 (*n* = 32) for the ACG prototypes. Team 10, using T340, found decarbonated EC/TC values of: 0.347 ± 0.026 for the SRM as shown in the table, and 0.385 for prototype filter ACG00192 (no uncertainty stated). Team 12, using TLT, analyzed three prototypes (ACG00686, ACG00688, and ACG00689) for an average EC/TC value of 0.443 ± 0.003 (*n* = 4); a single measurement of the bulk SRM spread on a quartz filter gave 0.416. Results for all three teams point to a slight enrichment of EC in the prototype filter compared to the SRM, on average by a factor of 1.066 ± 0.025. Such an enrichment is not to be ignored, but clearly it is trivial compared to the differences among clusters as shown in Fig. 2.

##### Kinetic Modeling

Two methods, TOK and Ch(Cr)K, explicitly use an empirical kinetic model to fit the observed carbon loss rate function, as a means to partition the total carbon into classes of differing reactivity. According to this model, the most refractory component is taken to be EC. The first method utilizes thermal oxidation and the second, chemical oxidation, but both fit the reaction rate function as a sum of exponentials. The more rapid isothermal oxidation of the TOK method provides sufficient data to estimate the EC component as an intercept, but the slower chemical oxidation of Ch(Cr)K yields only an imprecise estimate of a long lived component, and one is forced to take the residual carbon at the time of the longest exposure (406 h) as an upper limit measure of EC. Given unlimited time, it might be interesting to consider how long it would take the Ch(Cr)K process to attain an intercept comparable to the EC result of the more vigorous Ch(N3) process (hot, concentrated HNO_3_). The ratio of the EC results for the two procedures is 0.638, equivalent to an extension of just 0.648 half lives of the longer lived component. The total time to reach the EC level of the Ch(N3) procedure would then be 1.053 half-lives, and further reaction time might, in principle yield still smaller EC/TC values for this chemical oxidation technique, perhaps approaching the result of the thermal kinetic oxidation (TOK) technique. The practical problem is that the estimated half life of the longer lived component is 1003 h, so *44 days* would be required to approach the EC/TC result of the Ch(N3) procedure! This exercise in arithmetic is meant to highlight the kinetics perspective in attempts to estimate the “true” (asymptotic) value for EC—particularly in the practical application and interpretation of the strictly chemical oxidation techniques.

Kinetics considerations, of course, underlie many of the EC methods, which were developed through comprehensive investigations of the effects of time and temperature. Cases in point are the T340 and T375 methods. The former [15] showed also the quantitative impact of pyrolysis rates on the balance between premature soot loss and charring The impact of the chemical matrix on reactivity in the application of the latter method [26, 30] has special relevance to the intercomparison data in Table 2. This method has the distinction of having the largest number of teams represented in the exercise, and it is one of the simpler methods applied here, having no hybridization with “wet” chemistry or optics. Three of the four results received were within 10 % of one another, whereas the fourth was more than a factor of two higher. Kinetics was suspected to be a factor, for although the prescribed OC oxidation time (at 375 °C) was 24 h, Team 6 used 12 h, indicating that their tests showed it to be equivalent to the longer exposure period. After completion of the intercomparison, however, it was learned that these tests had been performed on SRM 1650, diesel particulate matter, rather than SRM 1649a.

##### Contrasts between identical or similar methods

Finally, in this method-based perusal of EC results, we consider: (1) results from different teams using the same method, (T375, TOT, Ch(N2)T, T340), and (2) results derived from two sets of superficially similar methods, (Ch(N4), Ch(N3)) and (TOT, TOR). The t-statistic was used to test for significant differences between pairs of results in Table 2. For those cases where the “same” method was applied by different participants there was no significant difference among the T375 results of Teams 2, 3, 4; but as noted above the EC/TC result for Team 6, where a shorter oxidation period was used, was significantly greater than the others (*p* = 0.015, 2-sided test). The pairs of results for Ch(N2)T (Teams 1, 7) and for T340 (Teams 7, 10) were quite comparable; while the somewhat greater difference for the TOT result pair (Teams 8, 18), was marginally significant (*p* = 0.04).

On the other hand, the two pairs of “similar” methods gave significantly different results: EC/TC by TOR was significantly greater than that given by TOT (*p* = 0.009); and EC/TC by Ch(N4) was significantly greater than that given by Ch(N3) (*p* = 0.019). This is important, because it provides an opportunity to gain some insight regarding the *underlying causes* of such differences. In fact, the TOT-TOR dilemma has been recognized for some time. One might think, that since both techniques have a built-in mechanism for pyrolysis correction, both would be correct, and therefore necessarily self-consistent. We are faced with the question as to just what methodological differences may be responsible for the fact that TOR often gives nearly twice as much EC as TOT. Two stand out: (1) pyrolysis monitoring by reflectance vs transmission, and (2) rather different temperature cycles. (The cycles are similar in that both begin with an anoxic thermal volatilization stage that can induce destructive decomposition and charring, and both conclude with a thermal oxidation stage for residual (charred) organic matter and EC.)

A recent comprehensive study of the two methods addressed these questions [6]. The key findings of the study are that both of the above procedural differences are consequential, but that the more important difference is the higher first (He) stage peak temperature of TOT (850 °C) compared to TOR (550 °C). It was suggested that matrix effects (high temperature catalytic oxidation by certain metal oxides) could result in premature loss of EC in the TOT procedure. Although not mentioned in the report, a contrasting possibility would be the survival of partially charred and polymeric organic material in He at 550 °C, followed by post split-time coevolution of such organic carbon with EC after the switch to oxygen.

The difference between the two acid digestion procedures using hot, concentrated nitric acid [Ch(N3), Ch(N4)] was somewhat surprising. The explanation may lie in the comments of Team 13, who noted that the results of Ch(N4) may tend to be high because of the absence of post-oxidation rinses to remove residual organic matter.

### 4.3 Isotopic (^14^C) Speciation

Intercomparison data for ^14^C in SRM 1649a are presented in Table 3, in four segments. Results are expressed in terms of the reference state “modern” (*f*_M_ = 1), which is artifactually defined by reference to the NBS/NIST SRM 4990B, as explained in Sec. 2.3.1. The actual, observed values for *f*_M_ have been adjusted for decay from the time of collection (1976–1977) to the time of measurement. (Adjustments were quite small because of the long half life of ^14^C, 5730 years.) Teams are identified parenthetically following the respective methods in the first column of the table. Method descriptions and literature references are given in Sec. 3.4. Data in the first segment of the table (“total carbon”) reflects the average isotopic composition of the SRM carbon. The following segments give *f*_M_ values for elemental and organic carbon classes, as well as values for eight individual PAH. The remarkable isotopic heterogeneity of this material is evident; it is an indication of widely disparate contributions from fossil and biomass carbon sources to the individual chemical fractions.

To give some perspective to the relative fossil and biomass carbon contributions to the several chemical fractions, the data have been transformed to indicate the approximate biomass carbon relative concentrations. This is given by *f*_C_ (last column) which refers to the fraction of “contemporary” carbon, where contemporary carbon is defined in terms of the ^14^C content of the living biosphere at the time of sampling. (The *f*_C_ values are given strictly to assist in interpretation of the results; the measured reference values, which are included in the certificate of analysis, must be expressed in terms of *f*_M_.) *f*_C_ is derived from *f*_M_ by taking into account the biospheric ^14^C enhancement factor from atmospheric nuclear testing; at the time of collection for SRM 1649a this factor was approximately 1.35. See Sec. 2.3.2 for further discussion of the “bomb” effect and some additional assumptions involved in the interpretation of the biomass carbon fraction.

Looking at the last column of Table 3, we see that on average, about 38 % of the SRM particulate carbon is derived from biomass. In contrast to this TC biomass carbon fraction, which is by definition *f*_C_ *averaged over all carbon species*, individual chemical fractions vary dramatically. While the aliphatic fraction derives almost entirely from fossil carbon, the aromatic fraction shows a substantial biomass component (13 % on average). Isotopic diversity within this (aromatic) fraction is strikingly exhibited, however, with individual compounds in the PAH sub-fraction being greater than 90 % fossil in origin.

Some further insight may be gained by considering isotopic-mass balance. It is clear that this *cannot be achieved* from the carbon fractions thus far measured, as indicated in the biomass fractions (*f*_C_) in the table. Although the average (total carbon) biomass contribution is 38 %, all of the measured fractions have smaller values; so there is necessarily a biomass component that has not yet been accounted for. The situation is not so bad for the organic fractions. The biomass contribution to the total extractable carbon^10^—derived from the *f*_M_ value in the SRM certificate of analysis—is 24 %, which is compatible with the range of values seen for polar, aromatic, and aliphatic carbon in the table. Looking deeper in the partition of organic species, we see that the PAH compounds from the aromatic fraction are decidedly more fossil in isotopic composition. They range from roughly 94 % fossil for benzo(*ghi*)perylene to 97 % fossil for pyrene. All have a significantly greater biomass component, however, than the aliphatic fraction (98 % fossil carbon). The PAHs, of course, account for only a trace fraction of the carbon in the aromatic fraction. The larger biomass component of the aromatic fraction (13 %) is supported by data on the specific portions of the “unresolved complex mixture” (ucm) that interfered with an early attempt to determine ^14^C in individual PAH ([54]; see especially the *note added in proof*). Application of isotopic mass balance equations to the ucm that was isolated with the benzo(*ghi*)perylene peak gave a biomass carbon contribution of ≈ 15 %.

The question of the missing biomass carbon in the *non-extractable fraction* of SRM 1649a is important, and some initial considerations will be presented elsewhere [56]. If one assumes that the non-extractable fraction comprises EC and polymeric carbon sub-fractions, then using two-stage isotopic-mass balance equations, it can be shown that the biomass carbon contribution to the polymeric component is 47 % if the EC biomass fraction is 4.8 % as given by the “thermal oxidation/residue” method for EC-^14^C; or 92 %, if the EC biomass fraction is 11.3 % as given by the “chemical oxidation/residue” method for EC-^14^C. The difference makes a strong case for devising an experiment to determine ^14^C in non-extractable, polymeric fractions of the carbonaceous particles.

Finally, the biomass fractions of the PAHs raise some interesting questions. First, there is the similarity between *f*_C_ of the “thermal oxidation/residue” EC method (0.048) and those of the PAHs (0.028 to 0.064). This makes a compelling, though circumstantial argument that both may represent products of high temperature combustion. Secondly, the somewhat larger biomass carbon contributions to the heavier PAH [0.064 for benzo(*ghi*)perylene] seem to belie the conventional wisdom that the higher molecular weight PAH are necessarily products of fossil fuel combustion. Prior laboratory studies, however, comparing PAH patterns from flaming combustion of oak, pine, and paraffin fuels, support the observations here, demonstrating significant emissions of benzo(*ghi*)perylene from biomass burning (pine, but not oak) [54].

## 5. Conclusion and Summary

Cooperation among 18 teams of experts participating in an international comparison of Particulate Carbon species in SRM 1649a has resulted in the generation of important data on Total Carbon (certified value), Elemental Carbon (information values), and Isotopic Carbon (^14^C Speciation; reference and information values). The two-fold outcome of this exercise has been: (1) production of particulate carbon analytical data for the new (2001) Certificate of Analysis for SRM 1649a, and (2) generation of critical information linking details of analytical methodology to ranges of intercomparison data, especially for the ratio EC/TC, and linking isotopic speciation data with EC data and methodology. A byproduct of the work on EC/TC was comparative data on the two intercomparison materials: the (bulk) SRM, *per se*, and the prototype ACG filter RM, which had been prepared by resuspending the bulk SRM in air.

A number of observations, drawn from the critique of the EC/TC results vs methodology, are summarized in Appendix 1. Three of the key observations are: (1) The extremely broad range of results, obtained by expert teams working with a homogeneous reference material, reflect in part imperfectly understood/corrected methodological artifacts (chemical and mechanical losses, incomplete reaction, charring), but perhaps more significantly the fact that *environmental EC is not a pure substance*. Therefore, different methods tend to measure different characteristics of this very complex material, which may range from pyrogenic “soot” at the one extreme to lower temperature pyrolysis “char” at the other. (2) There is the possibility, however, of achieving method-specific (operational) *EC/TC reference values for specific reference materials* (such as SRM 1649a). This is supported by the fact that three distinctive methods, applied by multiple teams, gave excellent within-method consistency. (3) In the light of team comments on possible artifacts specific to their methods, and the distribution of intercomparison results, we see the possibility of deconfounding artifactual EC from intrinsic differences related to analysis of more or less refractory regions of the “black carbon spectrum.”

Isotopic (^14^C) speciation adds a new dimension to the understanding of methodological differences and sources of individual chemical species in an isotopically heterogeneous reference material or environmental sample (Fig. 1, Table 3). The quest for isotopic mass balance, and complete isotopic speciation, generates challenging questions when that balance has not been achieved. In the present case, the combination of ^14^C-EC and chemical (EC/TC) data by two significantly different EC methods [T375, Ch(Cr)K], led to a pair of estimates for the, as yet uncharacterized, polymeric fraction of the SRM. The resulting bounds for biomass carbon in the polymeric fraction were 47 % and 92 %, respectively, the latter figure being especially intriguing in view of work that has been done on natural biopolymers in atmospheric aerosol [57, 58, 59]. Equally interesting is the fact that the ^14^C speciation in EC, isolated by the refractory “soot carbon” technique found in cluster group-1, was fully consistent with the predominately fossil carbon origin of the individual PAHs. Isotopic consistency should be expected, as both PAHs and EC-soot are known products/tracers of flaming (or exploding) high temperature combustion.

## Figures and Tables

**Table 1:** Individual Results for Total Carbon (TC) in SRM 1649a

Method	Team	TC^a^ (*w*)	*u* (*w*)	*n*
A. Combust(TOT)	8	0.1753	0.0046	3
B. Combust(GCTCD)	18	0.1778	0.0014	39
C. Combust(NDIR)	9	0.1766	0.0017	15
D. Combust(CHN)	6	0.1769	0.0016	10
D. Combust(CHN)	3	0.1760	0.0019	4
A. Combust(TOT)	5	0.1745	0.0067	2
E. Combust(2step)	10	0.1760	0.0029	4

a Mass fraction (*w*, in kg/kg)

**Table 2:** Individual Results for Elemental Carbon in SRM 1649a

Row	Method	Team	EC/TC	*u*(EC/TC)	*N*
1	AETH	1	0.069	0.004	4
2	T375	2	0.074	0.002	≥2
3	T375	3	0.077	0.002	3
4	T375	4	0.079	0.005	3
5	TOK	5	*0.109*	*0.005*	9
6	T375	6	0.187	0.013	6
7	TOT	18	0.200	0.005	4
8	Ch(N1)T	7	0.224	0.013	5
9	TOT	8	0.258	0.014	3
10	Ch(N2)T	7	0.280	0.004	6
11	Ch(N2)T	1	0.283	0.022	3
12	Ch(N3)	9	0.292	0.017	5
13	T340	7	0.346	0.025	5
14	T340	10	0.347	0.026	4
15	TOR	11	*0.432*	*0.009*	3
16	TLT	12	0.438	0.006	5
17	Ch(Cr)K	13	0.458	0.025	9
18	T500_EGA	14	*0.500*	*0.010*	2
19	Ch(N4)	13	0.520	0.057	3

Notes

EC/TC: Italics denote measurements on ACG prototype filters.

Row-6: T375 oxidation time 12 h, rather than 24 h.

Row-7: TOT tabulated result is for the bulk SRM; filter (ACG) result [Team 5] is *0.210* (*u* = *0.007*, *n* = 7).

Row-8: Ch(N1)T result for small samples; possibility of particle loss.

Row-9: TOT tabulated result is for the bulk SRM; filter (ACG) result is *0.264* (*n* = 32).

Row-14: T340 tabulated result is for the bulk SRM; filter (ACG) result is *0.385* (*n* = 1).

Row-16: TLT tabulated result is the average of five results reported by Team 12. Separate results for the bulk SRM and filter (ACG) are 0.416 (*n* = 1) and *0.443* (*n* = 4).

Row-18: T500 tabulated result is upper limit, because of possible OC co-evolution; OC solvent removal result, *0.40*, is a lower limit, because of particle loss.

**Table 3:** SRM 1649a Isotopic (^14^C) Speciation

	*f*_M_ (modern-C)	*u*(*f*_M_)^a^	*f*_C_ (biomass-C)
Total Carbon (Team)			
Combustion-Manometry (16)	0.61	0.04	0.45
H_3_PO_4_-Combustion-Manometry (13)	0.505	0.003	0.374
Combustion-GC-CHN (17)	0.517	0.004	0.383
Elemental Carbon (Team)			
Thermal oxidation/residue (4)	0.065	0.003 (*n* = 3)	0.048
Chemical oxidation/residue (13)	0.153	0.002	0.113
Thermal kinetic oxidation/intercept (5)	0.038	0.012	0.028
Organic Fractions (Team)			
Polar Carbon (4, 15)	0.43	0.01	0.32
Aromatic Carbon (16)	0.17	0.04	0.13
Aliphatic Carbon (4, 15)	0.024	0.006	0.018
Individual PAH (Teams = 4, 15)			
Phenanthrene	0.0406	0.0049	0.0301
Methylphenanthracenes	0.0434	0.0057	0.0321
Fluoranthene	0.0637	0.0026	0.0472
Pyrene	0.0372	0.0022	0.0276
Benz[*a*]anthracene	0.0413	0.0037	0.0306
Chrysene/Triphenylene	0.0553	0.0030	0.0410
Benzofluoranthenes (b,j,k)	0.0842	0.0027	0.0624
Benzo[*ghi*]perylene	0.0864	0.0046	0.0640

a Poisson standard uncertainties (*u*) are given for all *f*_M_ values except the first EC datum, where *u* is based on replication (*n* = 3).

## Footnotes

1 Note that three data quality descriptors are used in NIST Certificates of Analysis [2, 12]. An NIST *certified value* is a value for which NIST has the highest confidence in its accuracy, in that all known or suspected sources of bias have been investigated or accounted for. *Reference values* are noncertified values that represent best estimates of the true values; however, these values do not meet the NIST criteria for certification and are provided with associated uncertainties that may reflect only measurement precision, may not include all sources of uncertainty, or may reflect a lack of sufficient statistical agreement among multiple analytical methods. An *information value* is considered to be a value that will be of use to the SRM customer, but which has insufficient information to assess its associated uncertainty or for which only a limited number of analyses were performed. (For the “particulate carbon” section of the SRM 1649a Certificate of Analysis, Tables 12 and 13, the limiting number of analyses is taken to be two. That is, when uncertainties were derived from replication (“type A” uncertainties), the results were designated information values unless the number of replicates (*n*) was three or more.)

2 Support for the earlier data for carbonate carbon came as a byproduct of the present intercomparison, from four pairs of replicate measurements of SRM 1649a TC, with and without carbonate removal, by Team 10. Results for TC (g/g) were 0.1735 (*u* = 0.0023), non-decarbonated; and 0.1760 (*u* = 0.0029), decarbonated. The difference is −0.0025 (*u* = 0.0037) g/g; hence no carbonate-C was detected, but the upper limit is consistent with the prior Information Value.

3 The stable isotope ^13^C has a role in source discrimination and the control of isotopic mass balance, but its importance in atmospheric studies is overshadowed by that of ^14^C, largely because it lacks the remarkable (fossil-biomass) source discrimination power of ^14^C. The combination of both isotopes can be useful in special cases, however, as in the discrimination of fossil-C sources from C_3_ and C_4_ biomass sources [17]. ^13^C did not constitute a major part of this SRM 1649a intercomparison, partly for the above reasons, and partly because of the stringent measures that would be required to minimize errors from isotopic fractionation.

4 Minor differences in TOT temperature profiles are found in different laboratories, using the same basic (“Sunset”) instrument described in Ref. [24]. For the NIST instrument used by Teams 5 and 18: the volatilization (He) profile had steps of 200 °C, 380 °C, 560 °C, and 900 °C; and the oxidation (5 % O_2_ in He) profile had steps of 600 °C, 700 °C, 850 °C, and 900 °C.

5 First stage thermal oxidation (340 °C) in the closed tube gave the possibility of trapping (rather than evacuating) the first-stage OC gases in the small sample AMS target preparation apparatus for those cases where ^14^C AMS was applied to *both* the OC_340_ and the EC carbon from the same sample. It is especially useful when the *T340* procedure is adapted for OC, EC ^14^C speciation [38].

6 Note that all uncertainties given in this manuscript are standard uncertainties (*u*). For those based on replication (“type A” uncertainties), corresponding expanded uncertainties (*U*) in the certificate of analysis for SRM 1649a are given by *t*(df, 0.025)*u* as recommended in the ISO guide for the expression of uncertainty [16].

7 See Appendix 1 for a summary critique of the EC/TC data and the methods employed.

8 The essentially boundless spectrum of organic functionalities and structural characteristics comprising BC [30, 55] makes the question of a single correct analytical result ill-posed. In contrast to a complete chemical and structural characterization of each sample, simpler thermal and chemical methods of analysis would be expected to reflect different aspects of the material, ranging from partially condensed pyrolysis carbon (char) to highly condensed pyrogenic structures (polyaromatic soot).

9 The caution against over-interpretation will be seen to be well advised by the subsequent discussion of sample and methodological artifacts, which occupies much of the remainder of this section. In fact, consideration of the comparative results in light of possible artifacts in the associated methods may be one of the most important outcomes of this broad intercomparison exercise.

10 Extractable carbon was not included in this intercomparison, but results for its mass fraction (different solvents) are given in Tables 11 and 12 of the Certificate of Analysis, and its *f*_M_ value appears in Table 13 [2].

## 6. Appendix 1. Critique of EC Methodologies and Results

Some tentative generalizations follow from the interlaboratory, intermethod study of EC/TC ratios in a single homogeneous reference material (and its prototypical filter offspring).

- The fact that EC is not a pure substance means that the more vigorous oxidizing methods (e.g., T375) are more suited to analysis of the highly resistant (refractory) component of EC (soot), and other, gentler methods of thermal and/or chemical oxidation may be considered appropriate for assaying the more labile component (char).
- The corollary is that we should not expect to find a single correct value for EC in SRM 1649a, nor a single correct method; and comparative results are likely to be both method- and matrix-dependent.
- Significant inter-team differences did *not* appear for three rather diverse methods [T375, Ch(N2)T, T340], however, in the absence of protocol deviations (Sec. 4.2.2). As a result, there is a good possibility that selected *method-specific reference values* can be established.
- The EC/TC ratio of the prototype filter RM appears to exceed that of the bulk SRM, but only to a small extent (≈ 6 % relative).
- Sensitivity to reaction time (kinetics) and thermal regime/cycle deviations is critical for certain methods. Exhaustive interlaboratory investigations of optimal values and tolerable deviations may be essential for method certification.
- Matrix effects and artifacts require special vigilance: especially premature EC oxidation, coevolution of refractory (polymeric) organic carbon in the EC (thermal oxidation) peak, excessive optical density, optical attenuation coefficient variations, char formation from natural polymers, and pyrolysis correction accuracy. Such artifacts may lie at the heart of the discrepancies among “similar” methods, such as those found in clusters 2 and 3 of Fig. 2.
- SRM 1649a has a significant potential for charring, presumably from high molecular weight biopolymers. Pyrolysis carbon (char) produced in the TOT procedure was found to be comparable to the EC component itself. This means that special attention may be needed for those procedures that do not monitor charring, and to the accuracy of the charring correction, for those procedures that do.
- Accurate results for very small (sub-mg) samples demand exhaustive attention to microheterogeneity of materials, plus procedural blanks and particle loss, especially for isotopic speciation at the 10 μg to 100 μg (C) level.
- Finally, there is the matter of chemical and geochemical isotopic consistency. A striking illustration is given by comparison of the ^14^C abundance of “soot” EC with that of other pyrogenic products such as the PAH (Table 3). See also Ref. [30] Sec. 3.2.

## 7. Appendix 2. Team Members

**Table:**

Team-1:	Slater
Team-2:	Hedges, Prentice
Team-3:	Gustafsson
Team-4:	Reddy
Team-5:	Currie, Kessler
Team-6:	Hartmann, Quinn
Team-7:	Marolf, Currie
Team-8:	Cary
Team-9:	Klinedinst, Klouda
Team-10:	Cachier
Team-11:	Chow, Watson
Team-12:	Puxbaum, Schmid
Team-13:	Masiello, Druffel
Team-14:	Kirchstetter, Novakov
Team-15:	Benner, Eglinton, Pearson, Reddy, Wise
Team-16:	Currie, Klouda, Wise
Team-17:	Klinedinst
Team-18:	Klouda
